# The Heart of the Thymus: A Rare Tale of Nonischemic Cardiomyopathy in the Absence of Myasthenia Gravis

**DOI:** 10.1002/ccr3.72120

**Published:** 2026-06-04

**Authors:** Manish Barman, Bassem Al Hariri, Tasneem Babiker, Mohamed Gadkarem, Joudi Alhariri, Ahmad Alharafsheh

**Affiliations:** ^1^ HMGH, Hamad Medical Corporation Doha Qatar; ^2^ College of Medicine, Weill Cornell Medicine Doha Qatar; ^3^ College of Medicine Qatar University Doha Qatar

**Keywords:** anterior mediastinal mass, heart failure, myasthenia gravis, nonischemic cardiomyopathy, paraneoplastic syndrome, thymoma

## Abstract

Thymomas are rare epithelial tumors of the anterior mediastinum known for their association with autoimmune and paraneoplastic syndromes, most notably myasthenia gravis (MG). Cardiomyopathy is an exceptionally rare paraneoplastic manifestation. Emerging case reports suggest that thymoma may exert systemic effects independent of MG, including direct or immune‐mediated myocardial injury. We report the case of a 54‐year‐old male with known nonischemic cardiomyopathy who presented with septic shock from pneumonia and cholecystitis, complicated by ventricular arrhythmias and decompensated heart failure. Imaging revealed a calcified anterior mediastinal mass, later diagnosed as a World Health Organization (WHO) type B thymoma. Notably, the patient lacked any clinical or serologic evidence of MG or other autoimmune syndromes. Cardiac magnetic resonance imaging (MRI) excluded myocarditis and takotsubo cardiomyopathy, while coronary angiography ruled out ischemic disease. Despite anticoagulation, a persistent left ventricular (LV) thrombus remained. The patient's cardiac dysfunction necessitated mechanical ventilation, vasopressors, and a multidisciplinary heart failure regimen. This case expands the phenotype of thymoma beyond the traditional paraneoplastic spectrum. It reinforces the need for heightened clinical suspicion and a multisystem diagnostic approach in cases of unexplained cardiomyopathy. Further investigation into immune‐mediated cardiac injury in thymoma is warranted.

## Key Clinical Message

Thymoma warrants consideration in unexplained nonischemic cardiomyopathy, even in the absence of myasthenia gravis (MG). This case illustrates a rare paraneoplastic link where cardiac MRI and biopsy excluded myocarditis and ischemia, suggesting a novel immune‐mediated pathway. Early recognition is vital to balance complex anticoagulation needs and cardiac stabilization with tumor management.

## 1 Introduction

Thymomas are neoplasms of thymic epithelial origin, typically arising in the anterior mediastinum. Although rare, they constitute the most common anterior mediastinal tumor in adults, with an incidence of 0.13 per 100,000 person‐years [1]. These tumors often remain asymptomatic and are detected incidentally. A hallmark of thymoma is its association with autoimmune syndromes, particularly MG, which occurs in up to 40% of cases [1].

Nonischemic cardiomyopathy (NICM) is a heterogeneous group of myocardial disorders characterized by impaired ventricular function in the absence of obstructive coronary artery disease. Etiologies include genetic mutations, viral infections, autoimmune mechanisms, and toxin exposures [2]. Cardiomyopathy as a paraneoplastic manifestation of thymoma, particularly in the absence of MG, remains a clinical enigma. This case underscores the potential for thymoma to exert systemic effects beyond classical neuromuscular involvement and highlights the diagnostic and therapeutic complexities when it coexists with cardiac pathology.

## 2 Case Report

### 2.1 Patient Background

A 54‐year‐old man with a history of nonischemic dilated cardiomyopathy (left ventricular ejection fraction [LVEF] 30%), chronic left ventricular thrombus, and moderate‐to‐severe mitral regurgitation presented with progressive dyspnea, orthopnea, and lower limb edema. A computed tomography (CT) coronary angiogram performed 5 months earlier ruled out ischemia but revealed a suspicious anterior mediastinal mass. The patient was lost to follow‐up.

Home medications included apixaban, bisoprolol, perindopril, and dapagliflozin. He had a known history of depression and a recently diagnosed iron deficiency anemia.

### 2.2 Clinical Presentation

The patient presented to the emergency department with septic shock secondary to pneumonia and probable cholecystitis, accompanied by acute decompensated heart failure. Physical examination revealed hypotension (85/50 mmHg), tachycardia (heart rate 120 bpm), delayed capillary refill (> 3 s), bilateral basal crackles on pulmonary auscultation, and elevated jugular venous pressure (9 cm). Abdominal examination was soft and non‐tender. Neurological examination, including assessment for ptosis, diplopia, and muscle strength, was normal.

Chest X‐ray confirmed cardiomegaly and pulmonary congestion. Laboratory studies showed leukocytosis (white blood cell count 18 × 10^9/L), elevated C‐reactive protein (CRP 250 mg/L), acute kidney injury (creatinine 180 μmol/L), and transaminitis (ALT 120 U/L, AST 110 U/L). D‐dimer was markedly elevated (18.5 mg/L FEU). Blood and urine cultures were negative.

Electrocardiogram (ECG) showed sinus rhythm with poor R wave progression and T wave inversions in anterolateral leads. CT pulmonary angiography ruled out embolism but detailed a 5.7 × 3.3 × 6.2 cm calcified anterior mediastinal mass, anterior and to the left of the aortic arch and main pulmonary artery. The mass demonstrated well‐defined margins and rim calcifications, with no radiological evidence of invasion into adjacent great vessels, pericardium, or sternum. Associated mediastinal lymphadenopathy was noted, but no pathologically enlarged lymph nodes were seen.

### 2.3 Hospital Course

Initial management in the intensive care unit included noninvasive ventilation, intravenous (IV) fluid resuscitation with 2 L of normal saline over 6 h, and IV vasopressors (noradrenaline at 0.1 mcg/kg/min, titrated to a mean arterial pressure > 65 mmHg). Empiric broad‐spectrum IV antibiotics (piperacillin–tazobactam 4.5 g every 8 h) were administered for 7 days. Bedside ultrasound confirmed acute acalculous cholecystitis, which was managed conservatively.

Echocardiography showed worsening biventricular function (LVEF 25%–30%), a persistent LV apical thrombus, and moderate‐to‐severe mitral regurgitation. Cardiac MRI ruled out myocarditis and takotsubo syndrome, confirming nonischemic cardiomyopathy.

Due to an inadequate anticoagulation response to apixaban, therapeutic IV unfractionated heparin was initiated, targeting an activated partial thromboplastin time (aPTT) of 60–80 s. After 5 days, heparin was switched to IV bivalirudin (0.15 mg/kg/h) following the development of heparin‐induced thrombocytopenia. The patient developed frequent premature ventricular complexes (PVCs) and nonsustained ventricular tachycardia, for which metoprolol was uptitrated and later IV amiodarone (loading dose 150 mg over 10 min, then 1 mg/min for 6 h) was introduced. The patient required intubation following an episode of flash pulmonary edema and received mechanical ventilation for 6 days.

### 2.4 Diagnosis of Thymoma

**FIGURE 1:**
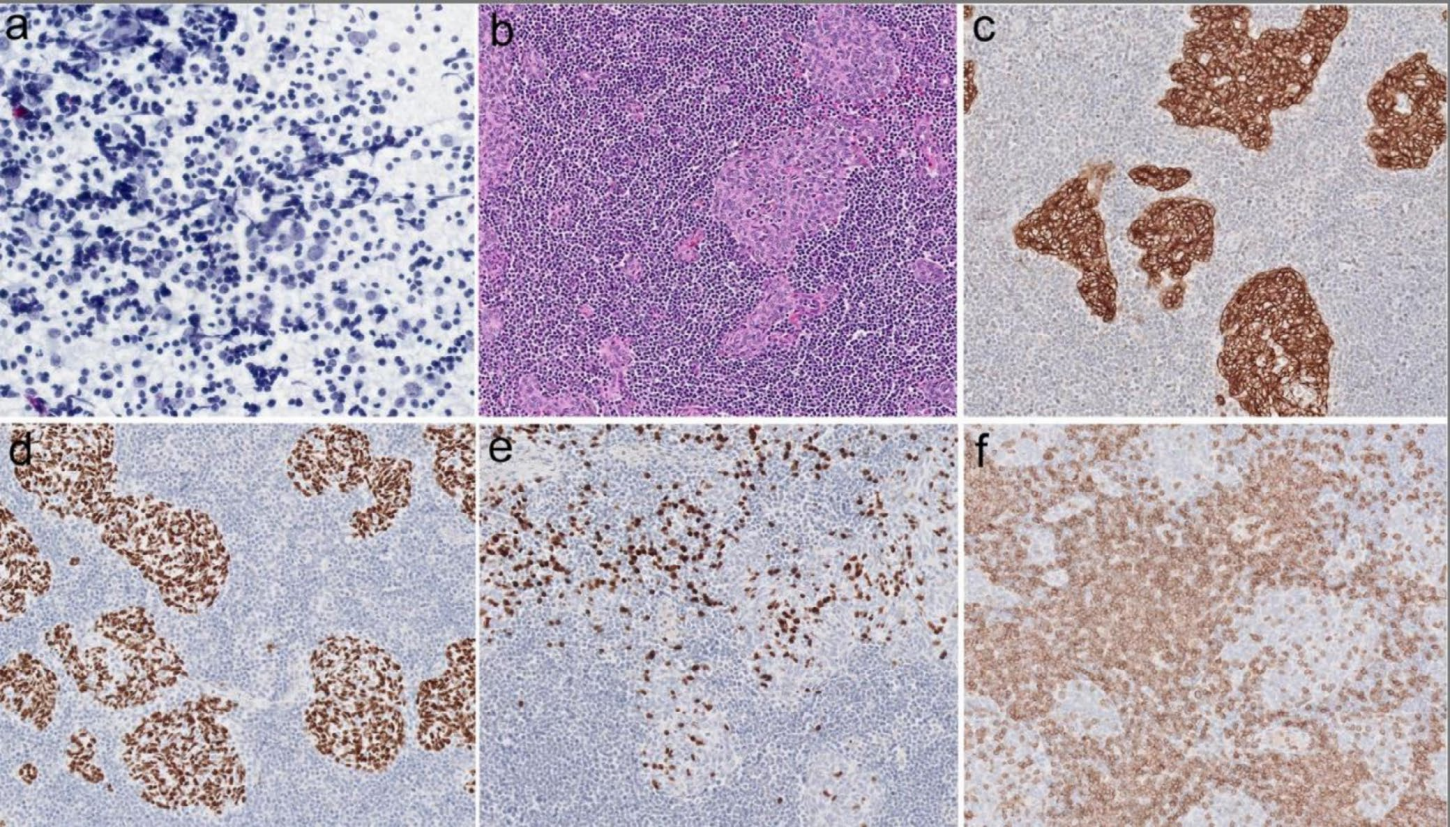
Histological and immunohistochemical slide images highlighting key features consistent with thymoma, WHO type B pattern and PAX8/CK5/6/p63 staining.

**FIGURE 2:**
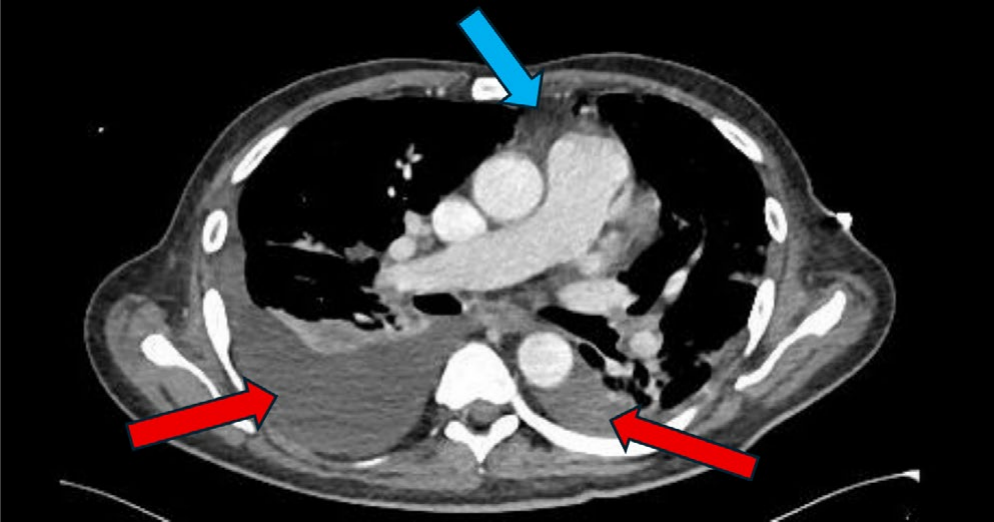
Thorax CT anterior mediastinal mass as described above, possibly representing thymus gland tumor (blue arrow), bilateral pleural effusion with associated lung collapse/consolidation more on the right side (red arrows).

A repeat contrast‐enhanced CT chest identified a well‐defined, calcified anterior mediastinal mass consistent with thymoma (Figure 2). CT‐guided biopsy confirmed a WHO type B thymoma. Histopathological analysis of the mediastinal mass biopsy revealed thymic epithelial fragments. Immunohistochemical staining was positive for PAX8, CK5/6, and p63, and negative for CD5 and CD117. These findings are consistent with a thymoma, most likely WHO type B1 or B2 (Figure 1). Final subtype confirmation would require surgical excision, given the limited tissue architecture in the biopsy specimen. No features of MG were observed clinically or serologically (negative acetylcholine receptor antibodies). A multidisciplinary team recommended a repeat biopsy and possible surgical resection pending cardiac stabilization.

## 3 Investigations Summary

- Hematology: hemoglobin 8.0 g/dL; ferritin 432 μg/L; transferrin saturation 12%.
- Cardiac markers: troponin‐T high sensitivity: 44 ng/L; N‐terminal pro‐brain natriuretic peptide (NT‐proBNP): 6361 pg/mL.
- Echocardiography: LVEF 30%; persistent apical thrombus; moderate‐to‐severe mitral regurgitation.
- CT Imaging: well‐defined hypodense mass in the anterior mediastinum with rim calcifications, measuring 5.7 × 3.3 × 6.2 cm. No evidence of local invasion. Bilateral pleural effusion with associated lung collapse/consolidation (Figure 2).
- Cardiac MRI: nonischemic biventricular cardiomyopathy without signs of inflammation or fibrosis.
- Autoimmune panel: antinuclear antibody, antineutrophil cytoplasmic antibodies, anti‐smooth muscle, anti‐mitochondrial, and anti‐liver–kidney microsomal antibodies all negative.
- Thymoma pathology: type B, nonmalignant, CD5/CD117 negative.

## 4 Discussion

This case highlights the rare concurrence of biopsy‐proven thymoma and NICM in the absence of MG or other autoimmune syndromes. Our patient's presentation and management contrast with and expand upon the existing literature. Traditionally, thymoma‐associated cardiac dysfunction has been linked to giant cell myocarditis or takotsubo syndrome, often in the context of autoimmune disorders [3, 7]. However, in our case, cardiac MRI definitively excluded myocarditis, and the clinical picture was not consistent with takotsubo physiology.

The pathophysiological mechanism remains elusive. The absence of systemic inflammation or serologic markers of autoimmunity suggests a possible novel paraneoplastic mechanism involving either subclinical autoimmunity or unrecognized immune modulators produced by thymoma cells [6]. This contrasts with cases where cardiac involvement is due to direct tumor infiltration, as reported by Priester et al. [4, 5], since our patient's lesion was noninvasive and anatomically confined. Our case aligns more closely with the report by Lim et al. [3], which describes severe biventricular failure linked to thymoma without MG, proposing tachycardia‐induced cardiomyopathy as a potential mechanism [8]. In our patient, arrhythmias were a consequence, not a primary cause, of the decompensated cardiomyopathy. In nonischemic cardiomyopathy, the heart becomes enlarged and pumps poorly (LVEF 30%). This creates blood stasis (sluggish flow), which naturally leads to thrombus formation even without a heart attack. Therefore, the thrombus is viewed as a complication of the pump failure, not the origin of it. However, cardiac MRI provided the critical evidence: It ruled out myocarditis and takotsubo cardiomyopathy. It confirmed the diagnosis of “non‐ischemic biventricular cardiomyopathy without signs of inflammation or fibrosis.”

The association between thymic neoplasms and myocardial dysfunction is a complex clinical entity with a historical lineage tracing back to Giordano and Haymond, who first established the triad of thymoma, MG, and giant cell myocarditis in 1944 [9].

The primary mechanism of myocardial injury is generally understood to be autoimmune, driven by a “spillover” of the immune dysregulation seen in MG. Kon et al. described the hallmark histological finding of giant cell myocarditis in these patients, characterized by an autoimmune reaction against striated muscle fibers in both the myocardium and skeletal muscle [10]. The molecular basis for this cardiotoxicity was further elucidated by Suzuki et al., who identified the critical role of anti‐Kv1.4 antibodies. Their review established that these striational antibodies are strongly associated with severe myocarditis and arrhythmias, such as QT prolongation, even in patients where classic MG symptoms are mild or absent [11].

The authors present a case of nonischemic cardiomyopathy concurrently with WHO type B thymoma that lacks the classical signs of MG, myocarditis, or takotsubo syndrome. Plausible hypothesis in this case hence could be summarized as follows: the traditional thymoma‐cardiac link, which involves MG‐associated giant cell myocarditis and anti‐Kv1.4 antibodies, was ruled out in this case by the absence of MG, the exclusion of myocarditis by cardiac MRI, and the noninvasive nature of the WHO type B thymoma. This suggests a novel paraneoplastic immune mechanism, likely driven by unrecognized immune modulators produced by the thymoma or subclinical autoimmunity [7] that induces nonischemic cardiomyopathy without the typical inflammatory change. While definitive proof is lacking, the most plausible pathophysiological explanation favored in this case is a novel, antibody‐independent paraneoplastic syndrome, wherein the dysregulated thymic epithelium secretes immunomodulatory factors that induce subclinical autoimmune‐mediated myocardial dysfunction, distinct from classic myocarditis.

Management of such patients is challenging. The opposing demands of stabilizing severe cardiac dysfunction and addressing the underlying thymoma create a clinical dilemma. In our case, the presence of active arrhythmias and a persistent LV thrombus significantly elevated the perioperative risk, delaying definitive surgical management of the thymoma. This contrasts with cases where the thymoma itself is malignant or invasive, necessitating urgent intervention. Furthermore, complications like thrombocytopenia added a layer of complexity to anticoagulation management. Based on our experience, we propose a staged management framework for similar cases: First, prioritize aggressive cardiac stabilization with guideline‐directed heart failure therapy and consider a trial of immunosuppression (e.g., corticosteroids) to target the suspected paraneoplastic driver, even in the absence of overt inflammatory markers. Second, defer tumor resection until there is objective evidence of cardiac stabilization or improvement (e.g., improved LVEF, resolution of life‐threatening arrhythmias, stabilization of ventricular thrombus). Third, undertake thymectomy with a tailored anesthetic and perioperative plan for cardiomyopathy, followed by long‐term cardiology–oncology follow‐up. This “cardiac‐first” approach prioritizes mitigating immediate life‐threatening risk over the theoretical oncologic benefit of early tumor removal. A prospective registry of thymoma patients with and without paraneoplastic syndromes may help elucidate this relationship and guide management protocols.

We would like to further elaborate on perioperative implications in this case. The presence of a persistent LV thrombus and active ventricular arrhythmias significantly increased the perioperative risk, necessitating that cardiac stabilization be prioritized over immediate thymoma resection. The severe cardiac dysfunction posed an immediate, life‐threatening risk, which outweighed the need for urgent intervention on the noninvasive thymoma. Although removing the tumor, the suspected source of immune modulators, might eventually improve the cardiomyopathy, this potential long‐term benefit was deferred due to the high immediate danger of perioperative cardiac death and thromboembolic events.

## 5 Conclusion

The authors present a case of nonischemic cardiomyopathy concurrently with WHO type B thymoma that lacks the classical signs of MG, myocarditis, or takotsubo syndrome occurring independently of MG or other classical paraneoplastic syndromes. This case contributes to emerging evidence that thymoma may induce cardiac pathology through mechanisms not yet understood. Clinicians should remain alert to atypical systemic manifestations of thymic tumors and adopt a multidisciplinary approach involving cardiology, oncology, and critical care in such complex presentations. Future research should focus on elucidating the immunopathological crosstalk between the thymus and the myocardium to identify potential biomarkers and targeted therapies.

## Author Contributions

**Manish Barman:** writing – original draft, writing – review and editing. **Bassem Al Hariri:** supervision, writing – original draft, writing – review and editing. **Tasneem Babiker:** writing – original draft, writing – review and editing. **Mohamed Gadkarem:** writing – original draft, writing – review and editing. **Joudi Alhariri:** writing – original draft, writing – review and editing. **Ahmad Alharafsheh:** writing – original draft, writing – review and editing.

## Funding

The authors have nothing to report.

## Ethics Statement

The study was conducted by the principles of the institutional ethical standards and national research committee.

## Consent

The patient provided oral and signed written consent to use his clinical materials in this study. Written informed consent was obtained from the patient for publication of this case report and any accompanying images.

## Conflicts of Interest

The authors declare no conflicts of interest.

## Data Availability

The data that support the findings of this study are available from the corresponding author upon reasonable request.
